# Synthesis and biological activity of α-l-fucosyl ceramides, analogues of the potent agonist, α-d-galactosyl ceramide KRN7000

**DOI:** 10.1016/j.bmcl.2010.04.079

**Published:** 2010-06-01

**Authors:** Natacha Veerapen, Faye Reddington, Gabriel Bricard, Steven A. Porcelli, Gurdyal S. Besra

**Affiliations:** a School of Biosciences, University of Birmingham, Edgbaston, Birmingham B15 2TT, UK; b Department of Microbiology and Immunology, Albert Einstein College of Medicine, Bronx, NY 10461, USA; c Department of Medicine, Albert Einstein College of Medicine, Bronx, NY 10461, USA

**Keywords:** CD1d, *i*NKT, Antigen, Ceramide, Lipid

## Abstract

Several l-fucoglycolipids are associated with diseases such as cancer, cystic fibrosis and rheumatoid arthritis. Activation of *i*NKT cells is known to lead to the production of cytokines that can help alleviate or exacerbate these conditions. α-Galactosyl ceramide (α-GalCer) is a known agonist of *i*NKT cells and it is believed that its fucosyl counterpart might have similar immunogenic properties. We herein report the synthesis of α-l-fucosyl ceramide derivatives and describe their biological evaluation. The key challenge in the synthesis of the target molecules involved the stereoselective synthesis of the α-glycosidic linkage. Of the methods examined, the per-TMS-protected glycosyl iodide donor was completely α-selective, and could be scaled up to provide gram quantities of the azide precursor **11**, from which a range of N-acylated α-l-fucosyl ceramides were readily obtained and evaluated for ex vivo expansion of human *i*NKT cells.

## Graphical abstract

The synthesis of α-l-fucosyl ceramide derivatives and their biological evaluation is described.

Both d- and l-fucose (6-deoxy galactose) are widely found in nature.^1^ Of interest, l-fucose is predominantly found in the α-configuration in the lipopolysaccharides (LPS) of Gram-negative bacteria and animal glycosphingolipids.^1,2^ With the exception of the monohexylceramide, α-l-fucosylceramide **1**, initially isolated from metastatic human adenocarcinoma,^3^ most fucosphingolipids are usually ceramide oligosaccharides.^2,4^ Many of these fucolipids are antigenic^5–7^ and play a role in tumour cell biology.^8–10^ Conversely, the synthetic glycolipid, α-galactosyl ceramide (α-GalCer)^11^ (**2**), is a strong agonist of *i*NKT cells when bound to CD1d, triggering the release of diverse cytokines, including both Th1 and Th2 cytokines.^12–14^ It is believed that the release of Th1 cytokines may contribute to antitumour and antimicrobial functions while that of Th2 cytokines may help alleviate autoimmune diseases^15–17^ such as multiple sclerosis^18^ and arthritis.^19^

**Figure:**
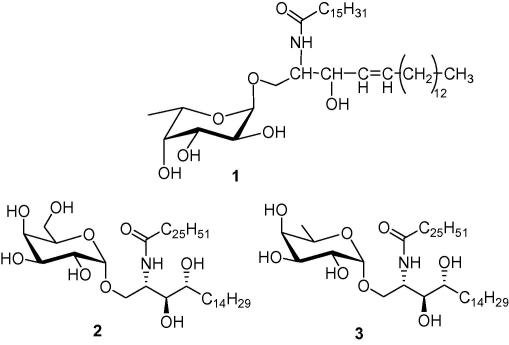

α-GalCer and its derivatives have proved to be and remain invaluable tools in understanding the functioning of CD1d and *i*NKT cells in a wide range of immune responses. Crystal structures of the bound α-GalCer–CD1d complex^20–22^ showed that the hydroxyl group at C-6 in the sugar head is not crucial for binding to the T-cell receptor (TCR) as opposed to C-2, C-3 and C-4. Hence, the α-d-fucosylceramide **3**^23^ was shown to be a potent inducer of IFN-γ in mice in vivo, while a slightly different derivative, synthesised by Motoki et al.^24^ showed strong lymphocytic proliferation stimulatory effects in vitro in mice.

While there are a few examples describing the synthesis of α-l-fucosylceramide **4b**^25^ and other derivatives in the literature,^26,27^ their biological activities are yet to be fully explored. As part of various ongoing studies, and because of the aforementioned reasons, we have synthesised compounds **4a**–**4d** for biological evaluation and comparative studies.

**Figure:**
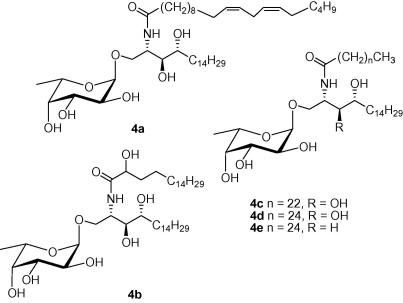

Fan et al.^25^ reported the synthesis of compound **4d**, while Okamoto et al.^27^ reported that of compound **4e**. Both groups obtained their target compounds as an α, β mixture (3:1 ratio) from different glycosyl donors and acceptors (Scheme 1). On the other hand, in the original reported synthesis of the fucosylceramide **1**, only the α-anomer was isolated when another ceramide acceptor was used under different reaction conditions.

We recently reported the synthesis^28^ of α-GalCer and other derivatives where we used *N*-iodosuccinimide (NIS) and triflic acid (TfOH) as a promoter, and benzoate protecting groups on the sphingosine base. Along with the nature of the protecting groups on both the glycosyl acceptor and donor, the choice of promoter, as well as temperature and reaction times can affect the stereoselectivity of the glycosylation reaction. In this study, we proposed to investigate whether our glycosyl acceptor **7** and reaction conditions could offer a better α-selectivity in the case of l-fucose.

The phytosphingosine acceptor **7**^29^ was synthesised from (2*S*,3*S*,4*R*)-2-azido-1,3,4-octadecanetriol^30^ as described before. Given the previous success of a thiogalactoside in a similar glycosylation reaction with compound **7**^28^ we chose to use the thiofucosyl donor **6**, rather than compound **5**. The thioglycoside **6** was thus obtained from commercially available l-fucose after standard procedures, as previously reported.^31^

With both the acceptor and donor in hand, we then proceeded to the critical glycosylation reaction. Interestingly, NIS/TfOH activation of 1 g of the thioglycoside **6** (Scheme 2) in anhydrous CH_2_Cl_2_ at −78 °C afforded the glycosylated compound **8**, almost exclusively as the α-anomer (α:β ratio = 9:1), in 68% yield after 2 h. Our results show a definite improvement in selectivity from the previously reported syntheses. Because the formation of the α-anomer is favoured by the anomeric effect, we rationalise that the latter is a governing factor under our reaction conditions. Both the lower temperature and the different reactivity of the acceptor **7** could potentially influence the stereoselectivity of the glycosylation reaction. These factors will have to be more thoroughly investigated in a later study. Subsequent Zemplen’s deprotection of the benzoate protecting groups produced the azide intermediate in quantitative yields. Tandem hydrogenation of the azido group and hydrogenolysis of the benzyl ethers in methanol then produced the amine **9** as a white solid, which exhibited spectroscopic data consistent with the literature.^25^

In an attempt to improve the stereoselectivity and circumvent the sometimes problematic removal of the benzyl ethers by hydrogenolysis, we embarked on a different glycosylation route. Recently, the use of glycosyl iodide donors has been revived by Gervay-Hague’s group.^32,33^ They have demonstrated that glycosylation reactions employing glycosyl iodides and promoted by tetrabutyl ammonium iodide (TBAI) are generally quite stereoselective and fast. It has been hypothesized that TBAI catalyses the isomerization of the α-glycosyl iodide to the β-anomer,^34^ thereby leading to the formation of an α-glycoside. They have successfully adapted this strategy to the synthesis of α-GalCer^35^ and other α-fucosylglycosides.^36^ Furthermore, the replacement of the benzyl ethers with trimethylsilylethers (TMS) made Gervay-Hague’s synthetic strategy even more attractive. TMS protected sugars are easy to generate and their deprotection requires very mild acidic conditions, compatible with the glycosidic linkage.

The per-*O*-trimethylsilyl-α-l-fucosylpyranosyl iodide **10** was generated by the reaction of the per-*O*-tetramethylsilyl-α-l-fucose with one equivalent of iodotrimethylsilane^35^ and then added to the phytosphingosine acceptor **7** which was premixed with diisopropylethylamine (DIPEA) and TBAI (Scheme 3). After 2 days at room temperature, the solvent was evaporated and the TMS protecting groups were removed by treatment with an acidic resin in MeOH. Compound **11** was obtained as the α-anomer exclusively in an overall yield of 62%. The formation of the desired α-linkage was confirmed by the H-1 and C-1 signals in ^1^H and ^13^C NMR. Methanolysis, followed by hydrogenation of the azide then afforded compound **9**.

Finally, N-acylation with the fully saturated fatty acids, tetracosanoic acid (C24:0) and hexacosanoic acid (C26:0), was achieved via reaction of the corresponding acid chloride with the free amine **9** in a 1:1 mixture of THF and saturated sodium acetate solution (Scheme 3). Target compounds **4c** and **4d** were obtained as white solids after concentration of the organic phase and purification of the residue by flash chromatography. The spectroscopic data of the final compounds were consistent with the literature.^25^ While compound **4a** was obtained by heating amine **9** with the *N*-hydroxysuccinimide activated ester of C20:2 fatty acid in a mixture of pyridine: water (9:1) at 50 °C overnight, compound **4b** was obtained via dicyclohexylcarbodiimide (DCC) activated coupling using procedures described previously.^37,38^

To assess the biological activity of the α-l-fucosylceramides and compare these to α-GalCer (KRN7000, **2**), we assessed the ability of each compound to induce the expansion of *i*NKT cells in samples of human peripheral blood mononuclear cells (PBMC) during an eight-day in vitro culture.^39^ The results showed that both the percentages and absolute numbers of *i*NKT cells in cultures were increased by stimulation with α-l-fucosylceramides with C26:0 (**4d**) > C18:0 (OH) (**4b**) > C24:0 (**4c**). The α-l-fucosylceramide containing a C26:0 fatty acid (**4d**) was the most active of the fucosyl series, and stimulated *i*NKT cell expansions in some donors that approached those seen with the prototype *i*NKT cell agonist KRN7000 (**2**). In contrast, the α-l-fucosylceramide containing the C20:2 fatty acid (**4a**) was found to lack detectable *i*NKT cell stimulating activity in any of the donors tested (Fig. 1B). Representative profiles obtained by flow cytometry of cultures from one normal blood donor are shown in Figure 1A. This analysis was carried out with PBMC from four separate donors (Fig. 1B). Although, differences were observed for the levels of *i*NKT cell expansion between different donors, all donors responded significantly to two of the α-l-fucosylceramide analogues (**4b** and **4d**).

In summary, in the current study we have developed an efficient method for the synthesis of a series of biologically active α-l-fucosylceramides.^40^ The second method, employing the per-*O*-trimethylsilyl-α-l-fucosylpyranosyl iodide **10**, proved to be superior with a better α-selectivity and reasonably good yield in the glycosylation reaction. Given the marked difference in the stereochemistry of the α-fucosyl head group of these glycolipids compared to α-galactosyl group of strong *i*NKT cell activators like KRN7000, it is surprising that the compounds in the current series show such substantial activity. This would seem to further reinforce the notion that the TCR of *i*NKT cells, in spite of its relative limited variability, is nevertheless able to interact efficiently with a broad range of structurally diverse ligands. It is noteworthy that the C20:2 *N*-acyl variant of α-fucosylceramide (**4a**) showed no detectable *i*NKT cell stimulating activity, given that the α-galactosyl version of this compound is an extremely potent *i*NKT cell agonist.^41^ This suggests that modification of the carbohydrate moiety can significantly alter the influence of the lipid moiety of the ligand on CD1d presentation and *i*NKT cell responses. Although the mechanism for this remains unclear, it is an important consideration for synthetic strategies that seek to combine biologically active alterations of the carbohydrate and lipid moieties of *i*NKT cell ligands.

## Figures and Tables

**Figure 1:**
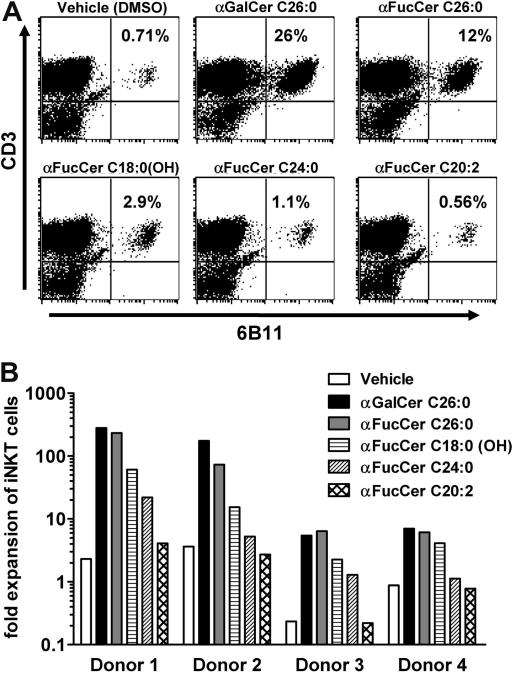
*Ex vivo expansion of human iNKT cells by α-l-fucosylceramides*. PBMC from four different donors were stimulated with the indicated glycolipids at a concentration of 250 nM in the presence of low levels of exogenous IL-2 and IL-7. At day 8, cultures were harvested and analysed by flow cytometry using monoclonal antibodies specific for CD3 and for the invariant TCRα chain expressed by *i*NKT cells (6B11). (A) Dot plots showing relative levels of CD3^+^ 6B11^+^*i*NKT cells are shown for one representative donor. Numbers in upper right quadrant indicate percentages of total lymphocytes that are *i*NKT cells. (B) Absolute numbers of *i*NKT cells in the cultures were determined by flow cytometry using fluorescent counting beads, and the values of *i*NKT cell fold expansion were determined by dividing by the input number of *i*NKT cells.

**Scheme 1:**
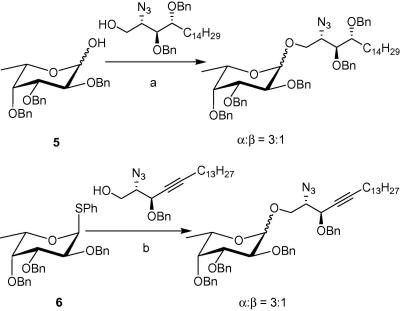
(a) Me_2_S, 2-Cl-pyridine, Tf_2_O, CH_2_Cl_2_; (b) dimethyl(methylthio)sulfonium triflate (DMTST), CH_2_Cl_2_, 0 °C-rt.

**Scheme 2:**
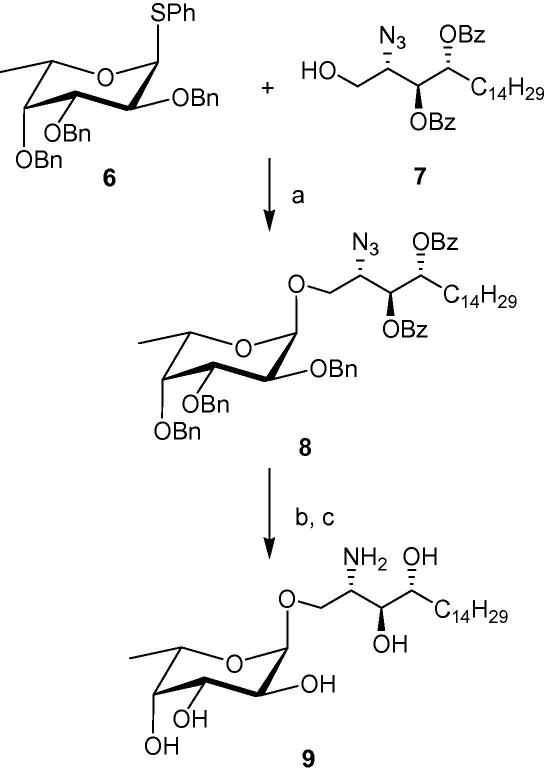
(a) NIS/TfOH, CH_2_Cl_2_, −78 °C to −20 °C, 68%; (b) NaOMe/MeOH, 92%; (c) H_2_, Pd, 76%.

**Scheme 3:**
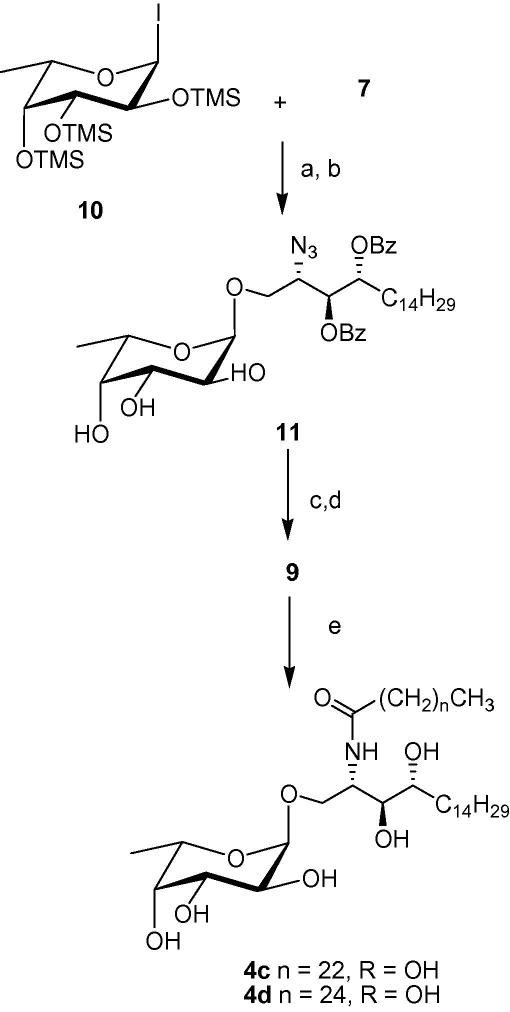
(a) TBAI, DIPEA, CH_2_Cl_2_, rt; (b) Dowex 50WX8-200, MeOH, rt, 62% over two steps; (c) NaOMe/MeOH, quantitative; (d) H_2_, Pd, MeOH, 80%; (e) C_25_H_51_COCl or C_23_H_47_COC1, THF, NaOAc, 78–80%.
